# Bi-Directional Pollution Characteristics and Ecological Health Risk Assessment of Heavy Metals in Soil and Crops in Wanjiang Economic Zone, Anhui Province, China

**DOI:** 10.3390/ijerph19159669

**Published:** 2022-08-05

**Authors:** Dun Wu, Hai Liu, Jian Wu, Ndhlovu kataza Nyasha, Wenyong Zhang

**Affiliations:** 1 Key Laboratory of Intelligent Underground Detection Technology, School of Civil Engineering, Anhui Jianzhu University, Hefei 230601, China; wudun@ustc.edu.cn (D.W.); wj18155922108@163.com (J.W.); nyashanash97@gmail.com (N.k.N.); 2 Public Geological Survey Management Center in Anhui Province, Hefei 230091, China; nftslh@gmail.com; 3 Exploration Research Institute, Anhui Provincial Bureau of Coal Geology, Hefei 230088, China

**Keywords:** bi-directional pollution, risk assessment, heavy metals, soil and crops, wanjiang economic zone

## Abstract

Understanding the extent of contamination, sources and various carcinogenic and non-carcinogenic risks associated with different heavy metals in soil-crop systems is crucial for the prevention of heavy metal pollution. A survey was undertaken to determine heavy metal concentrations and degree of pollution in soil-crop systems (rice, wheat, and corn) using various indices such as pollution factor (*CF*), geo-accumulation index (*I_geo_*), enrichment coefficients and transfer coefficient, and to determine the source of heavy metals pollution in the Wanjiang Economic Zone, Anhui Province, China. A total of 308 pairs of soil-crop samples were collected in this study, comprising 245 pairs of soil-rice samples, 53 pairs of soil-wheat samples, and 10 pairs of soil-corn samples. The concentrations of cadmium (Cd) and nickel (Ni) in the soil of the study area exceeded the national limitation of heavy metals in the soil of China (GB 15618-2018, *Soil Environmental Quality: Risk Control Standard for Soil Contamination of Agricultural Land. Ministry of Environmental Protection of China. Beijing. China*). The concentrations of copper (Cu), zinc (Zn) and lead (Pb) were also above the national limits to a lesser extent. All eight heavy metals (Cd, Cu, Ni, Pb Zn, arsenic (As), chromium (Cr), and mercury (Hg)) exceeded the background values in the study area. The enrichment coefficients of rice, wheat and maize to Cd, Cu and Zn were higher than those to other elements. On the basis of *I_geo_*, it can be indicated that the rhizosphere soil of rice was slightly polluted by Cd and Hg, while the concentrations of the other heavy metals were below the safety limits. The *CF* and pollution load index (*PLI*) indicated that the soil in the study area was heavily contaminated with heavy metals. A principal component analysis identified different sources of soil heavy metal pollution, that is, Cu, Pb, Zn and Cd from industrial sources, Cr and Ni from natural sources, and As and Hg from agricultural sources. The carcinogenic risk of heavy metals was related to the intake of crops. Residents in the study area ingest rice, wheat, and corn on a daily basis. On the basis this study, it is suggested that local governments should pay attention to the carcinogenic risk of heavy metals in rice.

## 1. Introduction

Soil is a key element of life on earth. Heavy metals in soil are related to the quality and safety of agricultural products and directly or indirectly affect human health. As such, the study of heavy metal levels has attracted extensive attention. With the development of intensified agriculture and the excessive use of various chemical pesticides and fertilizers, the accumulation of heavy metals in farmland soil has become a serious problem [1]. Heavy metals can enter the human body through a variety of pathways (such as soil intake, dust inhalation, skin contact with the soil, and food crops grown in contaminated soil) [2,3], causing cardiovascular, kidney, and nervous system diseases, or even cancer [3]. According to statistics, about 600 million people worldwide fall sick after consuming contaminated crops annually, and more than 420,000 people die [4]. Studies have displayed that heavy metals in crops are mainly derived from the soil environment in which they survive, and that their enrichment and accumulation are significantly different among different crops [5]. Therefore, it is of great practical significance to evaluate the harm potential to human health of heavy metals in soil and crops.

In recent years, a large number of studies [4,5,6,7,8,9,10,11,12,13,14,15,16,17,18] on soil-crop systems have been carried out, focusing on heavy metal content, pollution assessments and source identification, crop safety, and health risk assessments [4,5,6,7,8,9,10,11,12,13,14,15,16,17,18]. Crop varieties include rice [5,6,9,15], corn [12], wheat [12], vegetables [7,17], fruits [18] and tea [10], etc. Soil-crop pollution characteristics and health risks are still a current research focus. However, in previous studies, contaminated sites or typical farmland parcels were mainly used in most areas [4,5,6,7,8,9,10,11,12,13,14,15,16,17,18], and regional investigation work has been insufficient. Therefore, regional soil-crop heavy metals health risk assessments are conducive to optimizing and adjusting the structure of agricultural products to avoid food-borne hazards. In addition, the ability of various crops to absorb and accumulate heavy metals varies significantly. This results in more uncertainty in assessments of potential intake from different crop varieties through food consumption than the uncertainty associated with other exposure pathways, such as soil uptake and inhalation.

The Anhui section of the Yangtze River has superior agricultural resources, producing mainly rice, wheat, and corn [19,20]. Heavy metals such As Cd, Hg, As, Pb, iron (Fe), and manganese (Mn) in farmland soil and crops within Yangtze River exceeded the standard or were abnormal [20,21]. In the past, research on heavy metals in soil in the study area has mainly concentrated on single cities [19]. Little research has been conducted on the soil-crop system in the whole Yangtze River [22]. Few reports have emerged on the pollution levels, potential sources, ecological environment, and health risk of heavy metals in the soil-crop system, and there is a lack of a comprehensive understanding of the pollution status of heavy metals in the area (Anhui Section, Wanjiang Economic Zone). In view of this, the purpose of this study was to assess the pollution characteristics of heavy metals and their potential hazards to human health in the soil-crop system in the Yangtze River (Anhui Section). The research includes (1) the determination of heavy metal concentration in soil crops (rice, wheat, and corn); (2) evaluation of the pollution degree of heavy metals in the soil-crop system and determination of pollution sources; (3) a collaborative assessment of the ecological risks of heavy metals in soil-crop systems via different exposure pathways. This study was conducted to provide a theoretical basis and scientific reference for soil-crop heavy metal pollution assessments, remediation, and risk management.

## 2. Materials and Methods

### 2.1. Overview of the Study Area

The study area is located in the middle of Anhui Province, with geographic coordinates of 115°45′–119°23′ E and 29°34′–33°10′ N. The area comprises 75,800 km^2^ and includes 46 cities and counties. The main prefecture-level cities are Hefei, Wuhu, Anqing, Chizhou, Tongling, Maanshan, and Chuzhou. The study area has a subtropical, humid monsoon climate with an annual average temperature of 15.7~16.6 °C and annual average precipitation of 1500~1800 mm [22]. The area has a complicated landform type, with plains, hills, and low mountains as its main features. The terrain is high in the north and low in the south and generally inclines from southwest to northeast. There are many rivers and lakes, mainly distributed on both sides of the Yangtze River.

Magmatic rocks, sedimentary rocks, and metamorphic rocks are distributed in the study area, with complex geological structures and favorable ore-forming conditions. Metal and nonmetal mineral resources are abundant in reserves. More than 1900 large-, medium- and small-sized deposits have been discovered, of which the Tongling ore concentration area is an important part of the iron, copper, sulfur, and gold metallogenic belt in the middle and lower reaches of the Yangtze River [23]. The soil-forming parent materials in the study area are mainly river alluvial parent material, late Pleistocene loess parent material (vermicular network), laterite parent material, and bedrock weathered eluvial (diluvial) parent material. The soil types are mainly paddy and red soils. Land use types are mainly agriculture (including paddy fields, dry land, and garden plots) and forest land.

The rational allocation of light-heat-water-soil resources in the study area is advantageous for agricultural production. The grain crops in the region are mainly rice and wheat, while the cash crops are mainly cotton, rape, tea, hickory nut, tree peony bark, and fresh ginger.

### 2.2. Sample Collection and Testing

**Figure 1:**
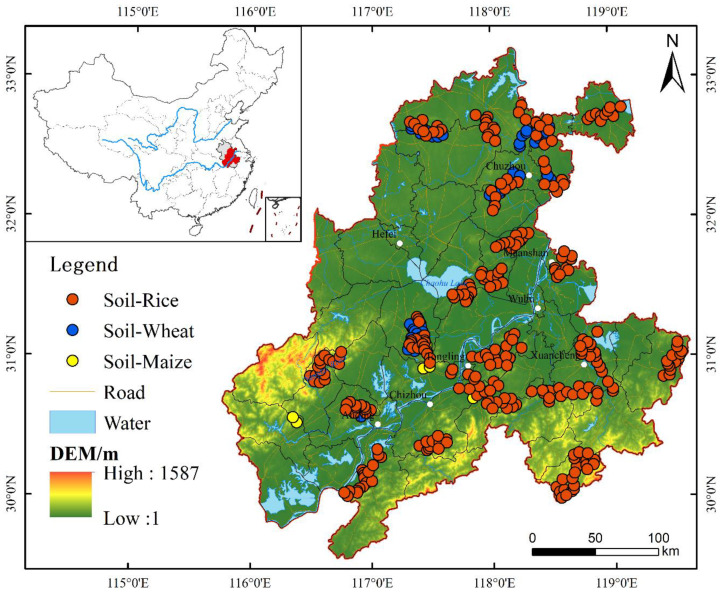
Location and distribution of soil-crop system sampling sites.

The collection of all soil-crop system samples not only considered the actual planting situation of agricultural products but also met the uniformity requirements of the sampling space. We focused on collecting rhizosphere soil samples. From July to August 2017, 308 pairs of soil-crop samples were collected (Figure 1), comprising 245 pairs of soil-rice samples, 53 pairs of soil-wheat samples, and 10 pairs of soil-corn samples. When collecting rhizosphere soil samples, the location of sample points was obtained by hand-held GPS navigation and positioning. When collecting crop samples, one needs to know the area and terrain of the whole field, as well as the growth of plants and the maturity-levels of seeds. At the same time, it is important to avoid collecting plants that are too big or small—especially those that suffer from diseases, pests, or mechanical injuries—or roadside plants. Soil samples were collected using a screw drill. According to the five-point quincunx method: four to five sampling points should be used; the periphery of the sampling points should be more than 1 m away from the edge of the ground; and each sampling point should be taken as a square of 60 cm × 60 cm, which is collected separately and combined into one sample with a sample weight of not less than 1000 g. In this respect, we collected mature ears of rice and wheat and mature complete corn cobs. The collected plant samples were dried, threshed, and peeled before being sent to the laboratory for preparation and analysis.

In accordance with the methods for Regional Geochemical Sample Analysis (DZ/T0279-2016), a test protocol consisting of the following analytical methods was selected. The concentrations of six elements (Cr, Ni, Cu, Pb, and Zn) were determined by X-ray fluorescence spectrometry (XRF). The concentration of Cd was determined by inductively coupled plasma mass spectrometry (ICP-MS). The concentrations of As and Hg were determined by atomic fluorescence spectrometry (AFS). The pH of the soil was determined by the ion selective electrode method (ISE).

According to Analysis Methods of Animal and Plant Samples for Eco-geochemical Evaluation (DZ/T0253-2014), inductively coupled plasma spectrometry (ICP-AES) and inductively coupled plasma mass spectrometry (ICP-MS) are the main methods for the detection of heavy metals in crops. These methods may be supplemented by atomic fluorescence spectrometry (AFS) and colorimetry.

### 2.3. Evaluation Method

In this study, the heavy metal pollution index, geo-accumulation index, and potential ecological risk index were used to evaluate the potential ecological risk level of heavy metal pollution in the soil-crop system. The enrichment coefficient method of heavy metals was used to evaluate the hazard of heavy metals to the ecological environment. Statistical methods such as principal component and correlation analyses were used to determine the sources of soil heavy metal pollution. The health risk assessment model was used to evaluate the health risk of heavy metals in the soil-crop system.

#### 2.3.1. Enrichment Factor Method (*EF*)

Enrichment factors are often used to judge the enrichment levels and sources of heavy metals in soil [24,25]. The calculation formula is as follows [26]:

(1) EF=CiCrefsampleCiCrefbackground

where *EF* is the enrichment factor of heavy metal elements in soil, *C_i_* is the concentration of heavy metals in soil, and *C_ref_* is the concentration of reference elements in soil. More generally, elements with stable geochemical properties during the supergene process are selected as reference elements, while elements such as scandium (Sc), Mn, titanium (Ti), aluminum (Al), and Fe are often selected as reference elements in the crust [27]. In this study, Fe was selected as a reference [28,29]. The enrichment coefficients are usually separated into five rankings [30,31]: *EF* < 2 for low enrichment, 2 ≤ *EF* < 5 for moderate enrichment, 5 ≤ *EF* < 20 for significant enrichment, 20 ≤ *EF* < 40 for high enrichment, and *EF ≥* 40 for extreme enrichment.

#### 2.3.2. The Geo-Accumulation Index

Geo-accumulation index (*I_geo_*) was put forward by German scholar Muller. It reflects the potential pollution degree of heavy metals in sediment or soil [32]. Its calculation formula is as follows:

(2) $$I_{geo}=\log_{2}\left(\frac{C_{n}}{1.5B_{n}}\right)$$

where *C_n_* is the concentration of a heavy metals in soil, *B_n_* is the background value of a heavy metal element in local soil, and the constant 1.5 is the correction coefficient of natural fluctuation of heavy metal concentrations during diagenesis [33]. The geo-accumulation index can be divided into seven levels [34]: *I_geo_* < 0 for no pollution; 0 ≤ *I_geo_* < 1 for no pollution to slight pollution; 1 ≤ *I_geo_* < 2 for moderate pollution; 2 ≤ *I_geo_* < 3 for moderate pollution to heavy pollution; 3 ≤ *I_geo_* < 4 for heavy pollution; 4 ≤ *I_geo_* < 5 for heavy pollution to extremely heavy pollution; *I_geo_ ≥* 5 for extremely heavy pollution.

#### 2.3.3. Pollution Factor (*CF*) and Pollution Load Index (*PLI*)

Pollution factor (*CF*) and pollution load index (*PLI*) can be employed to characterize the pollution degree of soil. The formula of pollution factor (*EF*) is as follows:

(3) $$CF=\frac{C_{metal}}{C_{background}}$$

where *C_metal_* is the concentration of the corresponding heavy metals in soil samples and *C_background_* is the background value of heavy metals. The *CF* can be divided into four rankings: *CF* < 1 for low pollution; 1 ≤ *I_geo_* < 3 for moderate pollution; 3 ≤ *I_geo_* < 6 for serious pollution; *CF* ≥ 6 for extremely serious pollution.

The *PLI* index reflects the general toxicity of heavy metals. Its calculation formula is as follows [35]:

(4) $$PLI=\sqrt[{n}]{CF_{1}\times CF_{2}\times CF_{3}\times \cdots \times CF_{n}}$$

where *n* is the number of heavy metals to be analyzed. Soil with *PLI* > 1 is considered to be polluted by heavy metals [36].

The single factor index method was used to evaluate the heavy metals pollution of crop seeds. The reference value is the safety value of the National Food Safety Standard Limits for Pollutants in Food (GB 2762-2017). The ratio of the concentration of elements in crop seed samples to the limits specified in the national food safety standards reflects the concentration of heavy metals.

#### 2.3.4. Enrichment Coefficient (Biological Concentration Factor)

The relationship between the concentration of elements in crops and the concentration of elements in the soil environment in which the crops live is an important factor by which to investigate whether crops can safely be grown in the soil. The biological concentration factor (*BCF*), which represents the distribution law of element concentration between soil and crops, is a basic index to estimate the quality of the soil environment. It has direct significance in evaluations of the ecological safety of soil and agricultural products. The calculation formula is as follows [37]:

(5) $$BCF=\frac{C_{p}}{C_{S}}$$

where *C_p_* is the concentration of a heavy metal element in crops and *C_s_* is the concentration of heavy metal elements in the soil.

#### 2.3.5. Potential Ecological Risk Assessment

On the basis of the interaction between the physical and chemical properties of heavy metals and the environment, Hakanson [38] proposed the concept of an ecological risk index to estimate the pollution degree of heavy metals and their potential ecological hazards. The formula is as follows:

(6) $$RI=\overset{n}{\underset{i=1}{\sum }}E_{r}^{i}=\overset{n}{\underset{i=1}{\sum }}\left(T_{r}^{i}\times P_{i}\right)=\overset{n}{\underset{i=1}{\sum }}\left(T_{r}^{i}\times \frac{W_{i}}{B_{i}}\right)$$

where *RI* is the comprehensive ecological risk index; $E_{r}^{i}$ is the potential ecological risk index of a certain heavy metal, i ; $T_{r}^{i}$ is the toxicity response coefficient of i ; $P_{i}$ is the enrichment coefficient of i ; $W_{i}$ is the measured value of i ; and $B_{i}$ is the background value of i . Studies have indicated that the toxicity response coefficients of Cu, As, Cd, Cr, Hg, Zn, Ni, and Pb are 5, 10, 30, 2, 5, 5, 5, and 5, respectively [39]. The potential ecological risk degree of a single factor can be divided into five rankings: $E_{r}^{i}<40$ for insignificant; $40\leq E_{r}^{i}<80$ for moderate; $80\leq E_{r}^{i}<160$ for heavier; $160\leq E_{r}^{i}<320$ for severe; $E_{r}^{i}\geq 320$ for serious. The total potential risk degree can be divided into four rankings: *RI* < 150 for insignificant; 150 ≤ *RI* < 300 for moderate, 300 ≤ *RI* < 600 for severe, and *RI* ≥ 300 for serious.

#### 2.3.6. Health Risk Assessment

Human health risk assessments are commonly used to quantify potential human health risks from exposure to certain heavy metals. In general, heavy metals present carcinogenic and non-carcinogenic risks to humans through direct oral intake, skin contact, and inhalation. In this study, the human exposure risk assessment model recommended by the U.S. Environmental Protection Agency (USEPA) was used to evaluate the carcinogenic and non-carcinogenic risks to adults and children in the study area. The calculation formula for average daily intake (*ADI*) of heavy metals in different exposure pathways is as follows:

(7) $$ADI_{doi}=\frac{C_{i}\times IngR\times EF\times ED}{\mathrm{BW}\times \mathrm{AT}}\;\mathrm{calculated}\;\mathrm{for}\;\mathrm{soil}\;\mathrm{and}\;\mathrm{crops}$$

(8) $$ADI_{ri}=\frac{C_{i}\times InhS\times EF\times ED}{\mathrm{PEF}\times \mathrm{BW}\times \mathrm{AT}}\;\mathrm{calculated}\;\mathrm{only}\;\mathrm{for}\;\mathrm{soil}$$

(9) $$ADI_{sc}=\frac{C_{i}\times \mathrm{SA}\times ABS\times EF\times ED}{\mathrm{BW}\times \mathrm{AT}}\;\mathrm{calculated}\;\mathrm{only}\;\mathrm{for}\;\mathrm{soil}$$

where $ADI_{doi}$ , $ADI_{ri,}$ and $ADI_{sc}$ are the *ADI* of direct oral intake, respiratory inhalation, and skin contact, respectively, in mg/(kg·d), and *C_i_* is the concentration of heavy metals in mg/kg. The meanings and values of other parameters are listed in Table 1.

Health risks are divided into non-carcinogenic and carcinogenic, among which the non-carcinogenic health risk index *HQ* or *HI* is expressed as follows:

(10) $$HQ_{ij}=\frac{ADI_{ij}}{R_{f}D_{ij}}$$

(11) $$HI=\sum {HQ}_{ij}$$

where *HQ_ij_* is the non-carcinogenic risk index of individual heavy metals and *HI* is the non-carcinogenic health risk index of multiple heavy metals. *R_f_D_ij_* is the reference dose in mg/(kg·d), presented in Table 2. When *HQ_ij_* or *HI* < 1, the non-carcinogenic health risk is not significant, i.e., it presents an acceptable risk. When *HQ_ij_* or *HI* > 1, there is a non-carcinogenic risk.

The carcinogenic risk index is calculated as follows:

(12) $$CR_{ij}=ADI_{ij}\times SF_{ij}$$

(13) $$TCR=\sum CR_{ij}$$

where *CR_ij_* is the single carcinogenic risk index, *SF_ij_* is the slope factor in mg/(kg·d), and *TCR* is the total carcinogenic risk of multiple heavy metals. When *CR_ij_* or *CR_ij_ <* 10^−6^, there is no carcinogenic risk, and when *CR_ij_* or *CR_ij_* is between 1 × 10^6^~1 × 10^4^, there is an acceptable risk.

### 2.4. Data Processing

The ArcGIS10.2 software (developed by Environmental Systems Research Institute, Inc., ESRI, Redlands, CA, USA) was employed to draw the sampling distribution map. Data processing and descriptive statistics were performed in the Origin 2021 software (Northampton, MA, USA), and SPSS 20.0 software (SPSS, Armonk, NY, USA) was used for correlation and principal component analyses to determine the sources of heavy metals in soil.

## 3. Results and Discussion

### 3.1. Heavy Metal Concentrations in Soil

Table 3 shows the statistical results of heavy metal concentrations in the rhizosphere soil of different crops in the study area. The average pH value of rhizosphere soil was less than 6.50, i.e., the soil was inadequately acidic. The variation ranges of Cr, Ni, Cu, Zn, Pb, Cd, As and Hg in rice rhizosphere soil were 23.20~242.20, 10.80~151.40, 13.50~1015.80, 30.30~416.90, 15.70~441.50, 0.08~2.45, 1.80~91.90 and 0.02~0.03 mg/kg respectively. The average values were Zn (83.62 ± 36.43 mg/kg) > Cr (70.45 ± 25.04 mg/kg) > Cu (43.71 ± 84.95 mg/kg) > Pb (37.22 ± 34.40 mg/kg) > Ni (28.80 ± 17.17 mg/kg) > As (11.67 ± 8.82 mg/kg) > Cd (0.34 ± 0.31 mg/kg) > Hg (0.09 ± 0.04 mg/kg). The variation ranges of Cr, Ni, Cu, Zn, Pb, Cd, As and Hg in wheat rhizosphere soil were 41.1~330.90, 17.50~300.49, 17.30~61.40, 43.50~137.30, 17.80~49.20, 0.068~0.086, 2.60~29.80 and 0.02~0.33 mg/kg respectively. The average values were Cr (88.78 ± 56.24 mg/kg) > Zn (72.94 ± 22.55 mg/kg) > Ni (44.96 ± 51.48 mg/kg) > Cu (31.00 ± 10.34 mg/kg) > Pb (27.33 ± 5.72 mg/kg) > As (11.50 ± 5.41 mg/kg) > Cd (0.15 ± 0.11 mg/kg) > Hg (0.05 ± 0.05 mg/kg). The variation ranges of Cr, Ni, Cu, Zn, Pb, Cd, As and Hg in maize rhizosphere soil were 28.30~65.00, 12.80~22.90, 17.00~248.70, 51.20~276.30, 17.40~39.70, 0.16~0.34, 2.57~22.13 and 0.02~0.08 mg/kg respectively. The average values were Zn (105.72 ± 68.81 mg/kg) > Cu (85.90 ± 77.12 mg/kg) > Cr (47.14 ± 12.03 mg/kg) > Pb (32.70 ± 15.46 mg/kg) > Ni (18.26 ± 3.20 mg/kg) > As (6.69 ± 5.71 mg/kg) > Cd (0.23 ± 0.07 mg/kg) > Hg (0.04 ± 0.02 mg/kg).

In comparison to the environmental background values of heavy metals in the soil in the study area [22], except for Cr and Ni in maize rhizosphere soil, the average concentrations of all heavy metals exceeded the environmental background values of the regional soil. In parallel, except for Cr and Ni in maize rhizosphere soil, the average concentrations of other heavy metals all exceeded the national environmental background values in China [46]. This shows that the rhizosphere soil of crops in the study area has been polluted by exogenous heavy metals. According to the risk screening value specified in the Control Standard of Soil Environmental Quality and Agricultural Land Soil Pollution Risk (Trial) (GB15618-2018), the over-standard rates of Cd and Ni in rice rhizosphere soil were 23.67% and 12.24%, respectively. Additionally, elements such as Cu, Zn and Pb in rice rhizosphere soil, Cr, Ni, Cu and Cd in wheat rhizosphere soil, and Cu and Zn in maize also exceeded the standard to different degrees.

The changing factors of heavy metal concentrations in rhizosphere soil of different crops were: Cu (194.33%) > Pb (92.44%) > Cd (91.72%) > As (75.53%) > Ni (59.63%) > Hg (51.05%) > Zn (43.56%) > Cr (35.55%) in rice rhizosphere soil, Ni (114.52%) > Hg (91.47%) > Cd (70.48%) > Cr (63.34%) > As (47.06%) > Cu (33.35%) > Zn (30.91%) > Pb (20.91%) in wheat rhizosphere soil and Cu (90.63%) > Zn (65.09%) > As (58.98%) > Pb (47.29%) > Hg (46.07%) > Cd (27.95%) > Cr (25.51%) > Ni (17.51%) in maize rhizosphere soil. The degrees of heavy metal concentrations in crop rhizosphere soil were as follows rice rhizosphere soil > wheat rhizosphere soil > maize rhizosphere soil. Among them, Cu in rice rhizosphere soil was abnormally strong, while Pb, Cd, As, Ni and Hg were strong variations; Ni in wheat rhizosphere soil was abnormally strong, while Hg, Cd, and Cr were strong variations. No abnormal variations were observed in maize rhizosphere soil, where only Cu, Zn and As were strong variations. This indicates that the change of heavy metal concentrations in crop rhizosphere soil in the study area were not only affected by geological background, but also by human activities, although needs to be further verified by source analysis [47]. The average concentration of heavy metals in the rhizosphere soil of farmland in Wanjiang Economic Zone was higher than that in the topsoil of farmland in northern Anhui Province [12] but lower than that in Tongling [42], where mining activities are intense. Compared with the average concentrations of heavy metals in crop rhizosphere soils in China’s Yangtze River Delta [47] and the Pearl River Delta [4], the average concentrations of Cr, Cd, Cu, Ni, and As in crop rhizosphere soils in Wanjiang Economic Zone were significantly higher. This may be directly related to the rapid urbanization which has occurred in this area. The rapid development of cities and towns has led to an increase of agricultural production and the massive application of fertilizers. Additionally, rapid economic development also leads to an increase in human engineering activities such as pit mining and smelting, which, in turn, lead to the enrichment of heavy metals [48,49].

### 3.2. Soil Heavy Metal Pollution Assessment

#### 3.2.1. Geo-Accumulation Index (*I_geo_*)

**Figure 2:**
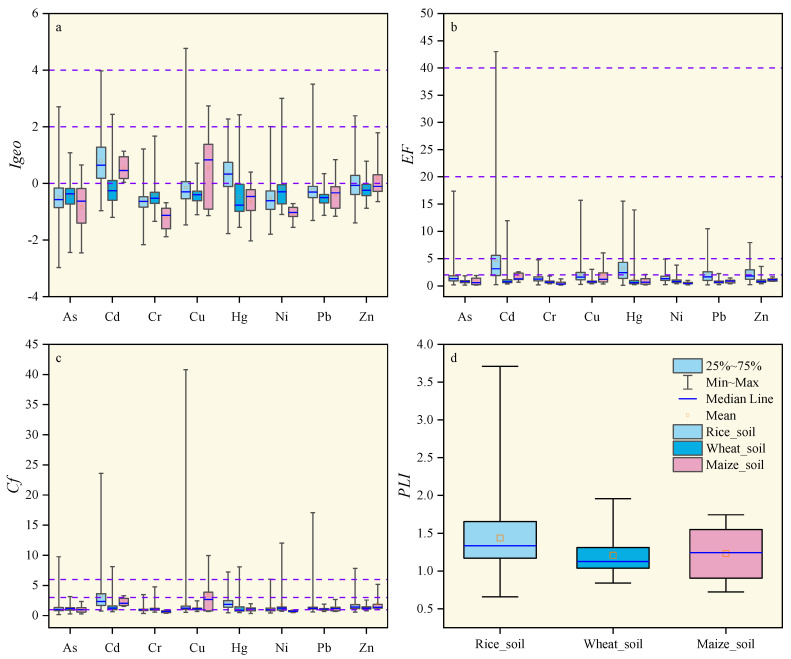
Boxplots of geo-accumulation index (*I_geo_*) (**a**), enrichment factor (*EF*) (**b**), contamination factor (*CF*) (**c**) and pollution load index (*PLI*) (**d**) for heavy metals in the study area.

The calculation results of the geo-accumulation index (*I_geo_*) of the study area are displayed in Figure 2a. The average values of heavy metals in rice rhizosphere soil, wheat rhizosphere soil and maize rhizosphere soil were: Cd (0.79) > Hg (0.33) > Zn (−0.03) > Cu (−0.15) > Pb (−0.22) > As (−0.48) > Ni (−0.52) > Cr (−0.63), Ni (−0.15) > Zn (−0.19) > Cd (−0.20) > Cu (−0.33) > Cr (−0.39) > As (−0.44) > Hg (−0.48) > Pb(−0.54) and Cu (0.55) > Cd (0.53) > Zn (0.19) > Pb (−0.39) > Hg (−0.66) > As (−0.83) > Ni (−1.06) > Cr (−1.19). Except for Cd and Hg in rice rhizosphere soil and Cu, Cd, and Zn in maize rhizosphere soil, the geo-accumulation index (*I_geo_*) of other elements was significantly below 1. According to Muller’s grading standard [32], Cd and Hg levels in rice rhizosphere soil and Cu, Cd, and Zn levels in maize rhizosphere soil indicated slight pollution, while other heavy metals did not indicate pollution. The *I_geo_* of heavy metals in rhizosphere soil was much lower than stated in [50], a study on urban and farmland soils in China.

#### 3.2.2. Enrichment Factor (*EF*)

The enrichment factor (*EF*) of rhizosphere soil in the study area is displayed in Figure 2b. The enrichment degree of heavy metals in rice rhizosphere soil, wheat rhizosphere soil, and maize rhizosphere soil was as follows: Cd (4.64) > Hg (3.21) > Zn (2.14) > Cu (2.06) > Pb (1.96) > As (1.71) > Ni (1.44) > Cr (1.34), Cd (1.09) = Hg (1.09) > Ni (0.98) > Zn (0.93) > Cu (0.82) > As (0.79) > Cr (0.77) > Pb (0.73) and Cu(2.17) > Cd (1.56) > Zn (1.13) > Hg (0.87) > Pb (0.81) > As (0.79) > Cr (0.52) > Ni (0.51). Except for Cd, Hg, Zn, and Cu in rice rhizosphere soil and Cu in maize rhizosphere soil, which were moderately enriched, all elements were low or not enriched. Luo et al. [51] found that Cd and Pb in urban soils in China were moderately enriched, while Hg was significantly enriched. Those authors suggested that human activities were the main reason for the enrichment of these heavy metals. Relevant research has shown that *EF* < 2 indicates that soil elements are enriched compared with the reference background. In contrast, *EF* > 2 indicates that soil-forming elements are enriched or influenced by some human input [52]. The enrichment factor in the process of natural soil formation is generally less than 2, so a higher *EF* value may point to human influence [53]. The average *EF* values of Cd, Hg, Zn, and Cu in rice rhizosphere soil and Cu in maize rhizosphere soil in the study area were greater than 2. The sources of these heavy metals can be attributed to human activities, while the rest of the elements in rhizosphere soil were likely of natural origin. The results show that the rhizosphere soil in the study area is polluted by heavy metals (Cd, Hg, Zn, Cu), mainly as a result of human activities.

#### 3.2.3. Contamination Factor (CF) and Pollution Load Index (*PLI*)

The calculation results of contamination factors in the study area are displayed in Figure 2c,d. The average contamination factors (CF) of heavy metals in rice rhizosphere soil, wheat rhizosphere soil, and maize rhizosphere soil were as follows: Cd (3.26) > Hg (2.12) > Cu (1.76) > Zn (1.57) > Pb (1.44) > As (1.24) > Ni (1.15) > Cr (1.02), Ni (1.80) > Cd (1.46) > Zn (1.37) > Hg (−1.33) > Cr (1.28) > Cu (1.24) > As (1.22) > Pb (1.06) and Cu (3.42) > Cd (2.26) > Zn (1.99) > Pb (1.26) > Hg (1.08) > As (1.03) > Ni (0.73) > Cr (0.68). The contamination factors of Cd in rice rhizosphere soil and Cu in maize rhizosphere soil indicated a considerable level of pollution, while the contamination factors of Cr and Ni in maize rhizosphere soil indicated an absence of pollution. Finally, all other elements indicated a slight level of pollution. The average pollution load indexes (*PLI*) of rice rhizosphere soil, wheat rhizosphere soil, and maize rhizosphere soil in the study area were 1.44, 1.21, and 1.23, respectively, and the pollution load index was greater than 1, which indicated that the soil in the study area had been polluted by heavy metals.

### 3.3. Potential Ecological Risk Assessment of Heavy Metals (*RI*)

**Figure 3:**
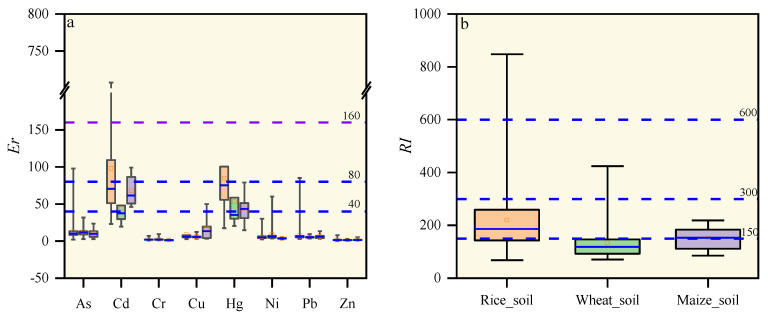
Boxplots of ecological risk (ER) (**a**) and potential ecological risk index (*PLI*) (**b**).

The basic statistical data of potential ecological risk factor (ER) and ecological risk index (*RI*) in the study area are displayed in Figure 3. Generally, Zn and Cr in the rhizosphere soil of the study area presented the lowest ecological risk, while Cd, As and Hg presented the highest. Except for the average ecological risk factors (ER) of Cd and Hg in all roots in the study area, which were greater than 40, all other elements were less than 40. The results indicated that except for Cd and Hg, all heavy metals presented low ecological risk. In contrast, the average ecological risk factors (ER) of Cd and Hg in rice rhizosphere soil were all greater than 80, which indicated that Cd and Hg in paddy soil presented considerable ecological risks. The high potential ecological risk factors of Cd are not only related to rice planting, but also to the enrichment of Cd caused by a number of agricultural fertilizers [48,49]. The average comprehensive potential ecological risk indexes (*RI*) of rice rhizosphere soil, wheat rhizosphere soil, and maize rhizosphere soil in the study area were 220.28, 133.53, and 151.74, respectively, which indicated that the heavy metals in the study area were at moderate pollution levels. Relevant research has indicated that the use of chemical fertilizers and pesticides can present high ecological risks in agricultural soils [54]. In eastern China, the high *RI* was due to the high Cd concentration in soil [55]; the same result was found in the Yangtze River Delta [47].

### 3.4. Traceability Analysis of Heavy Metals in Soil

#### 3.4.1. Correlation Analysis

**Figure 4:**
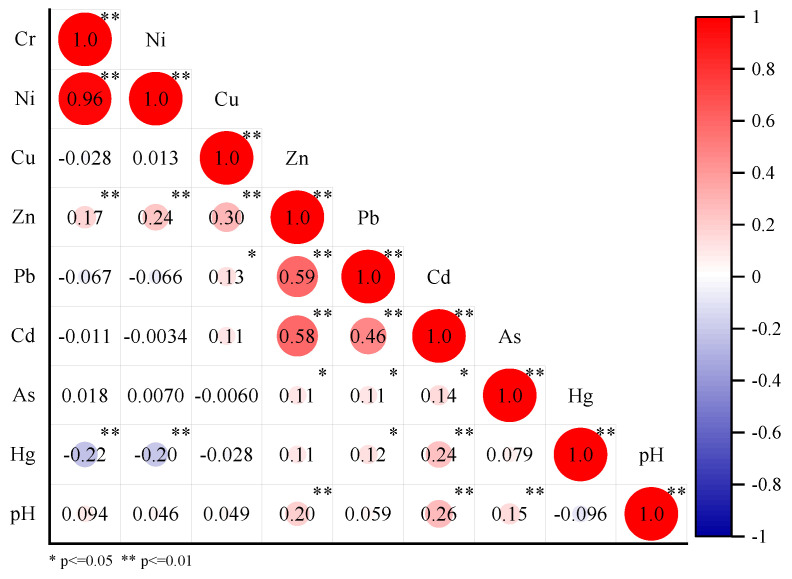
Plot of Pearson correlation coefficients.

The relationships among heavy metals can provide important information about the sources of such pollutants [56]. There was a very significant positive correlation between Cr and Ni in the soil of Wanjiang Economic Zone (*p* < 0.01) (Figure 4), with a correlation coefficient of 0.96, indicating that Cr and Ni may have similar sources. Zn, Pb, Cd, and Cu were positively correlated with each other (*p* < 0.01), likewise indicating that they may have similar sources. There was a significant negative correlation among Hg, Cr and Ni (*p* < 0.01), which indicated that Hg had different sources from Cr and Ni. In addition, there were no correlations between (i) Cr, Cd, and Pb, (ii) Ni and Cu, and (iii) Pb and As. This may indicate that these heavy metals had similar or different sources.

#### 3.4.2. Principal Component Analysis

The results of the principal component analysis suitability test indicated that the KMO value was 0.557, and the associated probability of the Bartlett spherical test was 0 (*p* < 0.01). The data were therefore considered to be suitable for principal component analysis. As the characteristic value was greater than 1, three principal components were extracted (see Table 4 and Table 5). The corresponding eigenvalues were 2.255, 2.082, and 1.084, and the cumulative contribution rate was 67.896%, which allowed us analyze most information about the eight aforementioned elements. In addition, the total cumulative contribution rate had not changed before or after the rotation, that is, the total amount of information had not been lost, indicating that these three factors can reflect most of the information contained in the data.

For factor 1 (PC1), the loads of Cu, Pb, Zn, and Cd were the highest; the variance contribution rate was 28.4192%. According to the correlation analysis, there were significant correlations among four elements: Cu, Pb, Zn, and Cd. Relevant research has indicated that industrial waste gas and automobile exhaust emissions are the main sources of heavy metal pollution in the atmosphere. Heavy metal elements in the atmosphere enter agricultural soil by wet and dry deposition, leading to the increase of Cd, Cu, Pb, and Zn contents in soil [57]. For example, the contribution rates of atmospheric deposition to Cd, Cu, Pb, and Zn levels in agricultural land in England and Wales were 52.90%, 38.93%, 77.73%, and 48.78%, respectively [57], while the contribution rates to agricultural soil in China were 34.83%, 0.02%, 84.85%, and 42.08%, respectively [51]. In addition, the massive application of livestock manure, especially pig and chicken manure in intensive farms, also leads to the accumulation of heavy metals, especially Cu and Zn, in soil [51,57]. The Wanjiang Economic Zone is a typical agricultural area in Anhui Province. However, with the rapid development of modern industry and the gradual emergence of township industrial parks, traffic and transportation are increasingly widespread. In addition, local residents breed livestock and poultry all year round, and there are several large-scale livestock and poultry farms. Additionally, the phenomenon of disorderly stacking of rural domestic garbage is quite serious. Therefore, according to the field investigation, the regional economy, and related research, it was concluded that Cd, Cu, Pb, and Zn mainly come from human activities, i.e., “industrial sources”.

The loads of Cr and Ni were the largest for PC2, with a variance contribution rate of 26.028%. Cr in the study area was largely absent. A correlation analysis indicated that Ni and Cr had a significant correlation (0.96) (*p* < 0.01). Relevant research has also indicated that the concentrations of Cr and Ni in soil are similar to those in parent materials, indicating that their presence was related to diagenetic components as opposed to human activities [51]. For example, in the Piemonte region of Italy [58] and the Mediterranean region of Europe [59], Cr and Ni in agricultural soils were mainly attributed to parent materials. Accordingly, Cr and Ni in PC2 were mainly attributed to “natural sources”.

PC3 described the combination of As and Hg; its variance contribution rate was 13.676%. The authors of [60] indicated that Hg and As are important components of pesticides, and that multiple applications of pesticides containing Hg and As lead to the enrichment of those elements in agricultural soil. Relevant research has indicated that about 5.5 × 10^7^ tons of chemical fertilizers flows into the soil every year in China [60]. In the present study, the research area is a typical agricultural area [42] in which large amounts of pesticides and fertilizers are applied every year. Therefore, it could be considered that As and Hg enter the soil and accumulate through the application of fertilizers. Accordingly, PC3 was considered to originate from “agricultural sources”.

### 3.5. Migration and Accumulation Characteristics of Heavy Metals in Crops

#### 3.5.1. Heavy Metal Concentrations and Pollution Assessments of Crops

Table 6 shows the statistical results of heavy metal concentration measurements of different crops in the study area. The variation ranges of Cr, Ni, Cu, Zn, Pb, Cd, As and Hg in rice were as follows: 0.08~0.66, 10.09~2.43, 1.22~8.17, 12.11~31.76, 0.03~0.14, 0.01~2.55, 0.01~0.26 and 0.0022~0.0325 mg/kg respectively; the average values were Zn (21.66 ± 1.16 mg/kg) > Cu (3.95 ± 1.16 mg/kg) > Ni (20.50 ± 0.41 mg/kg) > Cr (0.13 ± 0.06 mg/kg) ≈ Cd (0.13 ± 0.22 mg/kg) > As (0.1 ± 0.04 mg/kg) > Pb (0.06 ± 0.02 mg/kg) > Hg (0.0051 ± 0.003 mg/kg). The variation ranges of Cr, Ni, Cu, Zn, Pb, Cd, As and Hg in wheat were as follows: 0.11~0.38, 0.14~1.8, 3.69~9.92, 15.72~78.37, 0.05~0.33, 0.019~0.213, 0.03~0.064 and 0.001~0.013 mg/kg respectively; the average values were Zn (30.32 ± 10.33 mg/kg) > Cu (6.15 ± 11.32 mg/kg) > Ni (0.69 ± 0.48 mg/kg) > Cr (0.16 ± 0.05 mg/kg) > Pb (0.12 ± 0.05 mg/kg) > Cd (0.05 ± 0.04 mg/kg) > As (0.05 ± 0.01 mg/kg) > Hg (0.003 ± 0.0021 mg/kg). The variation ranges of Cr, Ni, Cu, Zn, Pb, Cd, As and Hg in maize were as follows: 0.12~0.30, 0.14~0.47, 3.67~8.42, 22.00~28.70, 0.05~0.31, 0.02~0.06, 0.03~0.05 and 0.0002~0.0009 mg/kg respectively; the average values were Zn (24.79 ± 2.28 mg/kg) > Cu (5.89 ± 1.50 mg/kg) > Ni (0.33 ± 0.10 mg/kg) > Pb (0.24 ± 0.07 mg/kg) > Cr (0.16 ± 0.05 mg/kg) > As (0.04 ± 0.01 mg/kg) > Cd (0.03 ± 0.071 mg/kg) > Hg (0.0006 ± 0.0002 mg/kg).

Except for the fact that the concentration of Pb in maize seeds slightly exceeded the limit of national food safety standard (GB2762-2017, *Maximum Levels of Contaminants in Foods, Ministry of Health of China. Beijing, China*), heavy metals in other crops were within the relevant limits. At a single sample point, 12.24%, 18.78%, 2.45%, and 1.22% of samples exceeded the national safety thresholds for Ni, Cd, As and Hg, respectively, in rice seeds. Additionally, Pb and Cd in wheat and maize also exceeded the standard.

The Cd concentration in rice seeds showed abnormal variation (174.67%), while Ni (81.41%) and Hg (58.30%) showed strong variation and all other pollutants moderate variation. In wheat seeds, Ni (68.74%), Cd (69.97%) and Hg (71.52%) indicated strong variation, while other elements indicated moderate variation. All of the tested heavy metal elements showed moderately strong variation in maize seeds. The above results indicate that there may be abnormal Cd enrichment in rice seeds.

#### 3.5.2. Bio-Concentration Ability of Crops for Heavy Metals in Soil

**Figure 5:**
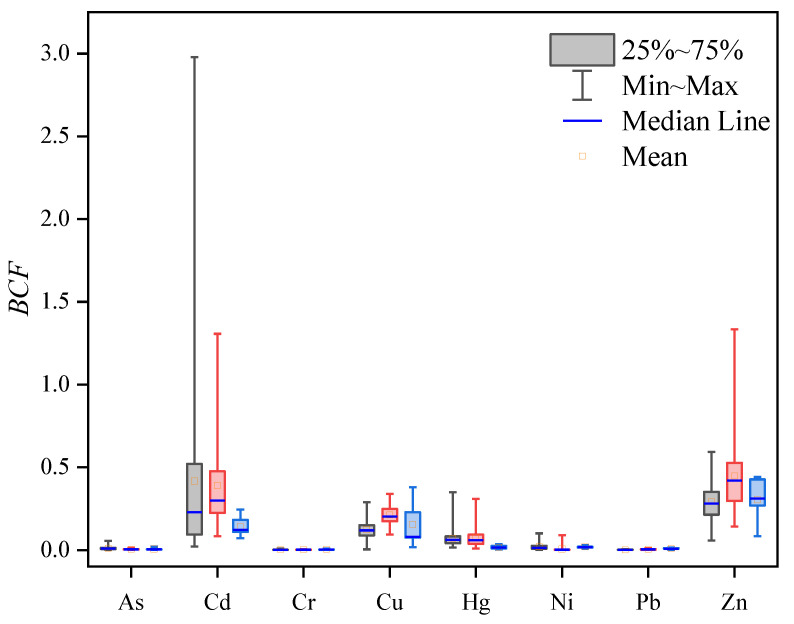
Boxplots of Bioconcentration factor for crops.

The bio-concentration factors (*BCF*s) of rice, wheat, and maize relative to soil heavy metals are displayed in Figure 5. The bio-concentration abilities of rice, wheat, and maize to heavy metals were as follows: Cd (0.4169) > Zn (0.2927) > Cu (0.1233) > Hg (0.0707) > Ni (0.0206) > As (0.0109) > Cr (0.0020) > Pb (0.0018), Zn (0.4483) > Cd (0.3900) > Cu (0.2130) > Hg (0.0771) > Ni (0.0222) > As (0.0049) > Pb (0.0049 46) > Cr (0.0020) and Zn (0.3041) > Cu (0.1546) > Cd (0.1413) > Ni (0.0183) > Hg (0.0177) > Pb (0.0088) > As (0.0066) > Cr (0.0038), respectively. The results indicated that the bio-concentration factors of Cd, Cu, and Zn in rice, wheat, and maize were high, while those of other elements were low, indicating that the bioavailabilities of Cd, Zn, and Cu in the study area were strong. Additionally, the Cd bio-concentration ability of rice was greater than those of wheat and maize, which was also an important reason for exceeding the standard of Cd in rice.

### 3.6. Health Risks Assessment of Heavy Metals

#### 3.6.1. Non-Carcinogenic Health Risk Assessment of Heavy Metals in Soil

**Figure 6:**
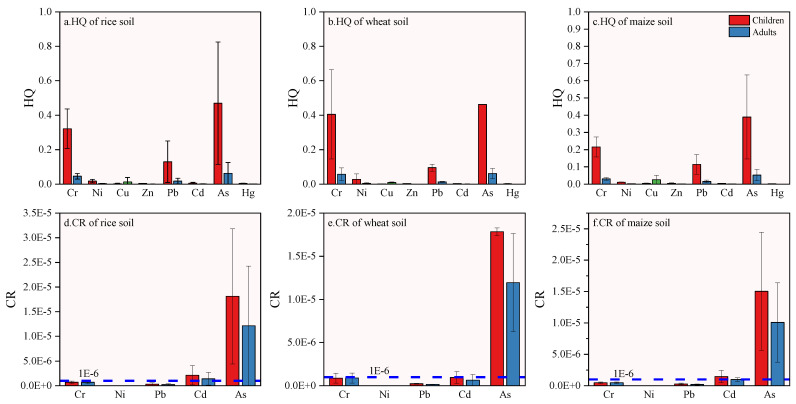
*HQ* (hazard quotient) and *CR* (carcinogenic risk) values for health risks for adults and children in different crops soil.

The single non-carcinogenic health risks index (*HQ*) and the total non-carcinogenic risks (*HI*) of heavy metals in soil through oral intake, skin contact, and respiratory inhalation for adults and children are displayed in Table 7 and Figure 6.

The *HQ* of adults and children exposed by three routes was as follows: oral intake > skin contact > respiratory inhalation. Thus, it was found that oral intake of heavy metals was the main exposure route of non-carcinogenic risk in the study area; this was consistent with other research results [62,63,64]. Eight heavy metal *HQ*s in rice rhizosphere soil, wheat rhizosphere soil, and maize rhizosphere soil were less than 1, which indicated that the health risk posed by individual heavy metals was not severe in the study area. From high to low, the *HQ*s of rice rhizosphere soil, wheat rhizosphere soil, and maize rhizosphere soil were as follows As > Cr > Pb > Ni > Cu > Cd > Hg > Zn (Figure 6a–c), which indicated that As, Cr and Pb were the main carcinogenic factors.

Except for children’s *HI* in wheat rhizosphere soil, which was greater than 1(1.01), all HIs were less than 1, indicating that there were no non-carcinogenic risks except with wheat rhizosphere soil. In addition, it was also found that the role of As in rice rhizosphere soil, wheat rhizosphere soil, and maize rhizosphere soil was the most obvious, accounting for 48.71%, 45.91%, and 50.90% of adults and 47.70%, 44.80%, and 50% of children, respectively.

#### 3.6.2. Health Risk Assessment of Heavy Metal Carcinogenesis in Soil

The carcinogenic risk indexes of As, Ni, Pb, Cd, and Cr in the rhizosphere soil of the study area are displayed in Table 8 and Figure 6.

The carcinogenic risk indexes (*CR*) of heavy metals for adults and children in different rhizosphere soils, from high to low, were both as follows As > Cd > Cr > Pb > Ni (Figure 6d–f). As had the highest carcinogenic risk, and its contribution rate to the comprehensive carcinogenic risk coefficient was over 85%, while the lowest risk was posed by Ni, i.e., less than 1%. According to the difference of carcinogenic risk between adults and children caused by heavy metals in soil, the carcinogenic risk of five heavy metals in rice rhizosphere soil, wheat rhizosphere soil, and maize rhizosphere soil to children in the study area was higher than that of adults. The comprehensive carcinogenic risk coefficients (*TCR*) for children and adults in three rhizosphere soils were as follows: 2.13 × 10^−5^, 1.45 × 10^−5^; 1.93 × 10^−5^, 1.36 × 10^−5^ and 1.73 × 10^−5^, 1.17 × 10^−5^ respectively. TCRs were within the maximum acceptable levels (10^−6^~10^−4^) recommended by USEPA. The results indicated that for all humans, considering single heavy metal or multiple heavy metal elements, the *CR* and *TCR* values of all samples were lower than the maximum acceptable levels as stated by USEPA, indicating that there was no obvious long-term health risk impact. Therefore, the lifetime carcinogenic risk of heavy metals in rhizosphere soil to children and adults was within the acceptable risk range.

#### 3.6.3. Non-Carcinogenic Health Risk Assessment of Heavy Metals in Crops

**Figure 7:**
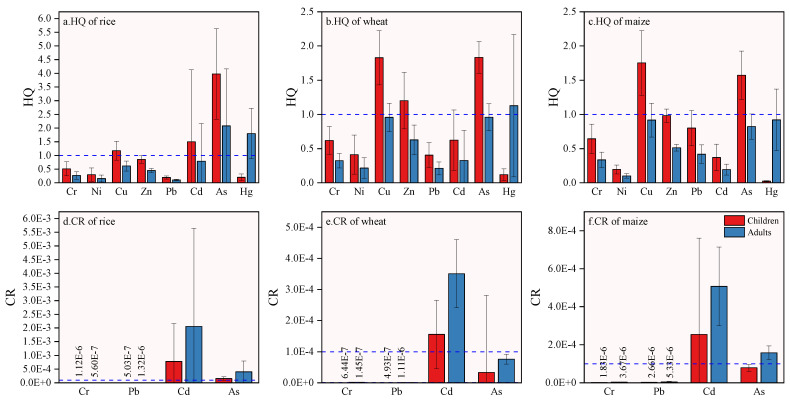
*HQ* (hazard quotient) and *CR* (carcinogenic risk) values health risks for adults and children in different crops.

The evaluation results of non-carcinogenic risk to children and adults caused by heavy metal intake in the study area are displayed in Table 9 and Figure 7. The *HQ* and *HI* of adults and children were both as follows: rice > wheat > maize. The non-carcinogenic risks of heavy metals in different crops to adults and children were different. For children, As in rice and wheat posed the greatest non-carcinogenic danger to children’s health, while Cu had the greatest influence in maize. For adults, As in rice and Hg in wheat and maize posed the greatest non-carcinogenic health risks to adults. In addition, the *HQ* values for children of Cu, Cd, and As in rice, Cu, Zn, and As in wheat, and Cu and As in maize were greater than 1, as were the *HQ* values for adults of As and Hg in rice and Hg in wheat (Figure 7a–c). In contrast, the *HQ* values of other elements in the three crops were less than 1.

The comprehensive hazard coefficients (*HI*) of eight heavy metals in rice, wheat, and maize for adults and children were all greater than 1, i.e., 6.26, 8.72, 4.75, 7.04, 4.22, and 6.34, respectively. The results indicated that the intake of rice in the study area could cause great harm to residents. Compared with adults, the comprehensive non-carcinogenic hazard coefficient of heavy metals in children was higher, i.e., 1.50, 1.39, and 1.48 times those for adults, respectively. This indicated that the non-carcinogenic risk of intake by children of crops in the study area was more serious, which is in line with results reported from other areas [15,65].

#### 3.6.4. Risk Assessment of Carcinogenesis of Heavy Metals in Crops

The evaluation and calculation results of the individual carcinogenic risk index (*CR*) and total carcinogenic risk (*TCR*) of adults and children due to heavy metal intake resulting from the consumption of crops in the study area are displayed in Table 10. The hazard coefficient of single heavy metal carcinogenic risk for adults and children caused by rice, wheat, and maize intake was follows: Cd > As > Cr > Pb (Figure 7d–f). The carcinogenic hazard index of four heavy metals ingested by adults and children through rice was much higher than those of wheat and maize.

The total carcinogenic risk to children and adults by ingesting rice, wheat, and maize were, respectively: 9.40 × 10^−4^ and 2.46 × 10^−3^, 1.90 × 10^−4^ and 4.29 × 10^−4^; and 3.37 × 10^−4^ and 6.75 × 10^−4^. The *TCR* of adults was significantly higher than that of children. All *TCR* values exceeded the maximum acceptable level (10^−6^~10^−4^) recommended by USEPA. It can be seen that the daily intake of rice, wheat, and maize by residents in the study area poses certain carcinogenic risks, and thus, the carcinogenic effects of heavy metals in rice on human health should be carefully studied.

## 4. Conclusions

In this paper, the pollution characteristics and ecological health risk posed by heavy metals in the soil-crop system in Wanjiang Economic Zone were studied. Compared with the concentrations of heavy metals in China’s soils elsewhere, Cd and Ni in the studied soils mainly exceeded the standard values. Cu, Zn and Pb also exceeded the standards to some extent, and all eight elements exceeded the background values for the study area. The concentrations of Ni, Cd, As and Hg in crop seeds exceeded the standard. The bio-concentration factors indicated that rice, wheat, and maize had higher enrichment coefficients for Cd, Cu and Zn, while other elements had lower bio-concentration factors.

The geo-eaccumulation index (*I_geo_*) indicated slight pollution from Cd and Hg in rice rhizosphere soil and Cu, Cd, and Zn in maize rhizosphere soil, while other heavy metals were largely absent. The enrichment factor (*EF*) indicated that Cd, Hg, Zn, and Cu in rice rhizosphere soil and Cu in maize rhizosphere soil were significantly enriched. The contamination factor (CF) and pollution load index (*PLI*) indicated that the soil in the study area had been polluted by heavy metals. The potential ecological risk assessment (RI) for heavy metals indicated that the soil in the study area was at a moderate pollution level.

A correlation analysis indicated that the heavy metals in the soil in the study area had similar or different sources. The results of a principal component analysis allowed us to identify three sources: Cu, Pb, Zn and Cd originated from “industrial sources”, Cr and Ni from “natural sources”, and As and Hg from “agricultural sources”.

The USEPA model was used to evaluate the health risks of soil and crops. There was no non-carcinogenic risk of heavy metals in the rhizosphere soil in the study area. The TCR results also indicated that the lifelong carcinogenic risk of heavy metals in the rhizosphere soil of the study area to children and adults was within an acceptable risk range. However, consuming crops grown in the study are will pose a non-carcinogenic risk due to the presence of heavy metals. In addition, the daily intake of rice, wheat, and maize by residents in the study area poses certain carcinogenic risks. In particular, the study area needs to focus on the carcinogenic effects of heavy metals in rice on human health.

## Figures and Tables

**Table 1:** Health risk assessment exposure parameters.

Parameter	Description	Unit	Value		References
			Children	Adults	
C_soil_	HM concentration in soil	mg/kg	This study	This study	This study
C_crops_	HM concentration in crops	mg/kg	This study	This study	This study
*EF*	Exposure frequency	days/year	350	350	[40]
*ED*	Exposure duration	year	6	30	[41]
IngS	Soil ingestion rate	mg/day	200	100	[40]
*IngR*_rice	Rice ingestion rate	g/day	198.4	389.2	[42]
*IngR*_wheat	Wheat ingestion rate	g/day	94.47	159.9	[16]
*IngR*_maize	Maize ingestion rate	g/day	259	389	[2,29]
*InhS*	Soil inhalation rate	m^3^/day	7.65	20	[43]
BW	Body weight	kg	16	60	[42]
PEF	Particle emission factor	m^3^/kg	1.36 × 10^9^	1.36 × 10^9^	[44]
AF	Adhesion factor of the skin	mg/cm	0.2	0.07	[40]
SA	Exposed skin area	cm^2^/d	2800	5700	[40]
ABS	Dermal absorption factor	Unitless	0.001	0.001	[40]
AT-noncarcinogenic	Average time	days	2190	10,950	[40]
AT-carcinogenic			25,550	25,550	[40]

Note: Equations (7)–(9) herein require the parameters in Table 1 to perform *ADI* calculations.

**Table 2:** Model parameters *RfD* and *SF* values.

Elements	*RfD*/(mg/kg·Day)			*SF*/(mg/kg·Day)		
	Ingestion	Inhalation	Dermal	Ingestion	Inhalation	Dermal
Cr	3.00 × 10^−3^	2.86 × 10^−5^	6.00 × 10^−5^	8.50 × 10^−3^	4.20 × 10^1^	NA
Ni	2.00 × 10^−2^	2.06 × 10^−2^	5.40 × 10^−3^	NA	8.40 × 10^1^	NA
Cu	4.00 × 10^−2^	4.02 × 10^−2^	1.20 × 10^−2^	NA	NA	NA
Zn	3.00 × 10^−1^	3.00 × 10^−1^	6.00 × 10^−2^	NA	NA	NA
Pb	3.50 × 10^−3^	3.52 × 10^−3^	5.25 × 10^−4^	8.50 × 10^−3^	NA	NA
Cd	1.00 × 10^−3^	1.00 × 10^−5^	1.00 × 10^−5^	6.10 × 10^0^	6.30 × 10^0^	NA
As	3.00 × 10^−4^	1.23 × 10^−4^	1.23 × 10^−4^	1.5 × 10^0^	1.51 × 10^1^	3.66 × 10^0^
Hg	3.00 × 10^−4^	8.57 × 10^−5^	2.10 × 10^−5^	NA	NA	NA

**Table 3:** The content of heavy metals in different crops soil.

Items	Parameter	pH	Cr	Ni	Cu	Zn	Pb	Cd	As	Hg
Rice soil (n = 245)	Minimum value	4.65	23.20	10.80	13.50	30.30	15.70	0.08	1.80	0.02
	Maximum value	8.38	242.40	151.40	1015.80	416.90	441.50	2.45	91.90	0.30
	Average value	6.50	70.45	28.80	43.71	83.62	37.22	0.34	11.67	0.09
	SD	0.98	25.04	17.17	84.95	36.43	34.40	0.31	8.82	0.04
	CV (%)	15.08	35.55	59.63	194.33	43.56	92.44	91.72	75.53	51.05
	Exceeded limits ^a^ (%)		0.00	3.27	12.24	0.82	2.45	23.67	2.45	0.00
Wheat soil (n = 53)	Minimum value	4.58	41.1	17.5	17.3	43.5	17.8	0.068	2.6	0.02
	Maximum value	8.03	330.9	300.9	61.4	137.3	49.2	0.846	29.8	0.33
	Average value	6.15	88.78	44.96	31.00	72.94	27.33	0.15	11.50	0.05
	SD	1.00	56.24	51.48	10.34	22.55	5.72	0.11	5.41	0.05
	CV (%)	16.24	63.34	114.52	33.35	30.91	20.91	70.48	47.06	91.47
	Exceed the limits ^a^ (%)		1.22	2.45	2.04	0.00	0.00	0.41	0.00	0.00
Maize soil (n = 9)	Minimum value	5.21	28.30	12.80	17.00	51.20	17.40	0.16	2.57	0.02
	Maximum value	7.32	65.00	22.90	248.70	276.30	69.70	0.34	22.13	0.08
	Average value	6.27	47.14	18.26	85.09	105.72	32.70	0.23	9.69	0.04
	SD	0.78	12.03	3.20	77.12	68.81	15.46	0.07	5.71	0.02
	CV (%)	12.40	25.51	17.51	90.63	65.09	47.29	27.95	58.98	46.07
	Exceed the limits ^a^ (%)		0.00	0.00	2.04	0.41	0.00	0.00	0.00	0.00
Risk screening value ^a^		6.50	250.00	70.00	50.00	200.00	100.00	0.40	30.00	0.50
Background value ^b^		5.85	69.40	25.00	24.90	53.20	25.90	0.10	9.40	0.04
Average of China (CNEMC, 1990)		6.80	57.30	24.90	20.70	68.00	23.50	0.08	9.60	0.04

^a^ Data obtained from MEE of China (Ministry of Ecology and Environment of China) (2018) [45]. ^b^ Data obtained from Chen et al. (2012) [22]. SD: Standard deviation; CV: variable coefficient.

**Table 4:** Characteristic values and accumulative contributions.

Component	Initial Eigenvalues			Extraction Sums of Squared Loadings			Rotation Sums of Squared Loadings		
	Total	% of Variance	Cumulative (%)	Total	% of Variance	Cumulative (%)	Total	% of Variance	Cumulative (%)
1	2.279	28.483	28.483	2.279	28.483	28.483	2.255	28.192	28.192
2	2.086	26.074	54.558	2.086	26.074	54.558	2.082	26.028	54.22
3	1.067	13.338	67.896	1.067	13.338	67.896	1.094	13.676	67.896
4	0.915	11.433	79.329						
5	0.812	10.149	89.478						
6	0.511	6.382	95.86						
7	0.297	3.713	99.573						
8	0.034	0.427	100						

**Table 5:** Results of principle component analysis.

Elements	Unrotated Component Matrix			Rotated Component Matrix		
	PC1	PC2	PC3	PC1	PC2	PC3
Cr	0.248	0.937	0.129	0.016	0.978	0.014
Ni	0.284	0.932	0.095	0.056	0.977	−0.015
Cu	0.338	−0.052	−0.706	0.414	−0.055	−0.664
Zn	0.891	0.013	−0.127	0.874	0.208	−0.06
Pb	0.733	−0.272	−0.046	0.776	−0.094	0.048
Cd	0.765	−0.235	0.126	0.781	−0.03	0.215
As	0.245	−0.068	0.576	0.191	0.061	0.596
Hg	0.211	−0.45	0.421	0.261	−0.334	0.495

**Table 6:** The content of heavy metals in different crops.

Items	Parameter	Cr	Ni	Cu	Zn	Pb	Cd	As	Hg
Rice grains (n = 245)	Minimum value	0.08	0.09	1.22	12.11	0.03	0.01	0.01	0.0022
	Maximum value	0.66	2.43	8.17	31.76	0.14	2.55	0.26	0.0325
	Average value	0.13	0.50	3.95	21.66	0.06	0.13	0.10	0.0051
	SD	0.06	0.41	1.16	3.48	0.02	0.22	0.04	0.0030
	CV (%)	49.83	81.41	29.34	16.08	28.75	174.67	41.46	58.30
	Exceeded limits ^a^ (%)	0.00	12.24	0.00	0.00	0.00	18.78	2.45	1.22
Wheat grains (n = 53)	Minimum value	0.11	0.14	3.69	15.72	0.05	0.019	0.03	0.0010
	Maximum value	0.38	1.8	9.92	78.37	0.33	0.213	0.064	0.0130
	Average value	0.16	0.69	6.15	30.32	0.12	0.05	0.05	0.0030
	SD	0.05	0.48	1.32	10.33	0.05	0.04	0.01	0.0021
	CV (%)	32.64	68.74	21.41	34.08	44.02	69.97	20.34	71.52
	Exceed the limits ^a^ (%)	0.00	5.31	0.00	0.41	0.41	1.63	0.00	0.00
Maize grains (n = 9)	Minimum value	0.12	0.14	3.67	22.00	0.05	0.02	0.03	0.0002
	Maximum value	0.30	0.47	8.42	28.70	0.31	0.06	0.05	0.0009
	Average value	0.16	0.33	5.89	24.79	0.24	0.03	0.04	0.0006
	SD	0.05	0.10	1.50	2.28	0.07	0.01	0.01	0.0002
	CV (%)	31.54	31.47	25.44	9.20	30.36	38.49	21.19	32.83
	Exceed the limits ^a^ (%)	0.00	0.00	0.00	0.00	3.27	0.41	0.00	0.00
Risk screening value ^a^		1.00	1.00	10.00	50.00	0.20	0.20	0.20	0.020

^a^ National standards for food safety (GB 2762-2017, Maximum Levels of Contaminants in Foods, Ministry of Health of China, Beijing, China) [61]. SD: Standard deviation; CV: variable coefficient.

**Table 7:** Risks of non-carcinogenic exposure to heavy metals in different pathways in different crops soil.

Evaluation Object		*HQ*								*HI*
		Cr	Ni	Cu	Zn	Pb	Cd	As	Hg	
Soil sample for rice	Children	3.22 × 10^−1^	1.74 × 10^−2^	1.32 × 10^−2^	3.39 × 10^−3^	1.30 × 10^−1^	5.22 × 10^−3^	4.70 × 10^−1^	3.60 × 10^−3^	0.96
	Adults	4.56 × 10^−2^	2.34 × 10^−3^	1.77 × 10^−3^	4.54 × 10^−4^	1.74 × 10^−2^	7.67 × 10^−4^	6.28 × 10^−2^	4.89 × 10^−4^	0.13
Soil sample for wheat	Children	4.05 × 10^−1^	2.72 × 10^−2^	9.38 × 10^−3^	2.96 × 10^−3^	9.53 × 10^−2^	2.34 × 10^−3^	4.63 × 10^−1^	2.26 × 10^−3^	1.01
	Adults	5.75 × 10^−2^	3.65 × 10^−3^	1.26 × 10^−3^	3.96 × 10^−4^	1.28 × 10^−2^	3.44 × 10^−4^	6.19 × 10^−2^	3.06 × 10^−4^	0.14
Soil sample for mazie	Children	2.15 × 10^−1^	1.11 × 10^−2^	2.57 × 10^−2^	4.28 × 10^−3^	1.14 × 10^−1^	3.61 × 10^−3^	3.90 × 10^−1^	1.85 × 10^−3^	0.77
	Adults	3.05 × 10^−2^	1.48 × 10^−3^	3.45 × 10^−3^	5.75 × 10^−4^	1.53 × 10^−2^	5.30 × 10^−4^	5.21 × 10^−2^	2.50 × 10^−4^	0.10

*HQ*: the non-carcinogenic risk index of a single heavy metal; *HI*: the non-carcinogenic health risk index of multiple heavy metals.

**Table 8:** Risks of carcinogenic exposure to heavy metals in different crops soil.

Evaluation Object		*CR*					*TCR*
		Cr	Ni	Pb	Cd	As	
Soil sample for rice	Children	7.01 × 10^−7^	6.99 × 10^−10^	3.25 × 10^−7^	2.13 × 10^−6^	1.81 × 10^−5^	2.13 × 10^−5^
	Adults	7.08 × 10^−7^	2.44 × 10^−9^	2.17 × 10^−7^	1.42 × 10^−6^	1.21 × 10^−5^	1.45 × 10^−5^
Soil sample for wheat	Children	8.83 × 10^−7^	1.09 × 10^−9^	2.39 × 10^−7^	9.53 × 10^−7^	1.78 × 10^−5^	1.99 × 10^−5^
	Adults	8.92 × 10^−7^	3.80 × 10^−9^	1.59 × 10^−7^	6.35 × 10^−7^	1.19 × 10^−5^	1.36 × 10^−5^
Soil sample for mazie	Children	4.69 × 10^−7^	4.43 × 10^−10^	2.86 × 10^−7^	1.47 × 10^−6^	1.50 × 10^−5^	1.73 × 10^−5^
	Adults	4.74 × 10^−7^	1.54 × 10^−9^	1.90 × 10^−7^	9.81 × 10^−7^	1.01 × 10^−5^	1.17 × 10^−5^

*CR*: the single carcinogenic risk index of a single heavy metal; *TCR*: the total carcinogenic risk of multiple heavy metals.

**Table 9:** Risks of non-carcinogenic exposure to heavy metals in different crops.

Evaluation Object		*HQ*								*HI*
		Cr	Ni	Cu	Zn	Pb	Cd	As	Hg	
Rice	Children	5.13 × 10^−1^	3.00 × 10^−1^	1.18 × 10^0^	8.59 × 10^−1^	1.97 × 10^−1^	1.50 × 10^0^	3.98 × 10^0^	2.02 × 10^−1^	8.72
	Adults	2.68 × 10^−1^	1.57 × 10^−1^	6.15 × 10^−1^	4.49 × 10^−1^	1.03 × 10^−1^	7.85 × 10^−1^	2.08 × 10^0^	1.80 × 10^0^	6.26
Wheat	Children	6.19 × 10^−1^	4.12 × 10^−1^	1.83 × 10^0^	1.20 × 10^0^	4.06 × 10^−1^	6.24 × 10^−1^	1.83 × 10^0^	1.19 × 10^−1^	7.04
	Adults	3.24 × 10^−1^	2.16 × 10^−1^	9.56 × 10^−1^	6.29 × 10^−1^	2.12 × 10^−1^	3.26 × 10^−1^	9.59 × 10^−1^	1.13 × 10^0^	4.75
Maize	Children	6.43 × 10^−1^	1.95 × 10^−1^	1.75 × 10^0^	9.82 × 10^−1^	8.00 × 10^−1^	3.71 × 10^−1^	1.57 × 10^0^	2.47 × 10^−2^	6.34
	Adults	3.36 × 10^−1^	1.02 × 10^−1^	9.17 × 10^−1^	5.14 × 10^−1^	4.19 × 10^−1^	1.94 × 10^−1^	8.22 × 10^−1^	9.21 × 10^−1^	4.22

*HQ*: the non-carcinogenic risk index of a single heavy metal; *HI*: the non-carcinogenic health risk index of multiple heavy metals.

**Table 10:** Risks of carcinogenic exposure to heavy metals in different crops.

Evaluation Object		*CR*				*TCR*
		Cr	Pb	Cd	As	
Rice	Children	1.12 × 10^−6^	5.03 × 10^−7^	7.85 × 10^−4^	1.53 × 10^−4^	9.40 × 10^−4^
	Adults	2.93 × 10^−6^	1.32 × 10^−6^	2.05 × 10^−3^	4.01 × 10^−4^	2.46 × 10^−3^
Wheat	Children	6.44 × 10^−7^	4.93 × 10^−7^	1.55 × 10^−4^	3.37 × 10^−5^	1.90 × 10^−4^
	Adults	1.45 × 10^−6^	1.11 × 10^−6^	3.51 × 10^−4^	7.60 × 10^−5^	4.29 × 10^−4^
Maize	Children	1.83 × 10^−6^	2.66 × 10^−6^	2.53 × 10^−4^	7.92 × 10^−5^	3.37 × 10^−4^
	Adults	3.67 × 10^−6^	5.33 × 10^−6^	5.07 × 10^−4^	1.59 × 10^−4^	6.75 × 10^−4^

*CR*: the single carcinogenic risk index of a single heavy metal; *TCR*: the total carcinogenic risk of multiple heavy metals.

## Data Availability

Not applicable.

## Sample Availability

Coal samples are not available from the authors.
