# Correction: Wu et al. Bi-Directional Pollution Characteristics and Ecological Health Risk Assessment of Heavy Metals in Soil and Crops in Wanjiang Economic Zone, Anhui Province, China. *Int. J. Environ. Res. Public Health* 2022, *19*, 9669

**DOI:** 10.3390/ijerph22091434

**Published:** 2025-09-15

**Authors:** Dun Wu, Hai Liu, Jian Wu, Ndhlovu kataza Nyasha, Wenyong Zhang

**Affiliations:** 1 Key Laboratory of Intelligent Underground Detection Technology, School of Civil Engineering, Anhui Jianzhu University, Hefei 230601, China; wudun@ustc.edu.cn (D.W.); wj18155922108@163.com (J.W.); nyashanash97@gmail.com (N.k.N.); 2 Public Geological Survey Management Center in Anhui Province, Hefei 230091, China; nftslh@gmail.com; 3 Exploration Research Institute, Anhui Provincial Bureau of Coal Geology, Hefei 230088, China

Xia Gao and Guojun Cai were removed as authors in the original publication [1]. The corrected Author Contributions statement appears here. The authors state that the scientific conclusions are unaffected. This correction was approved by the Academic Editor. The original publication has also been updated.

Author Contribution: D.W. and H.L. was responsible for conceptualization, methodology, and writing—original draft preparation; J.W. was responsible for investigation; N.k.N. and W.Z. was responsible for writing—review and editing. All authors have read and agreed to the published version of the manuscript.
